# The Role and Potential of Modern Radiotherapy in the Treatment of Metastatic Prostate Cancer

**DOI:** 10.3390/cancers17061045

**Published:** 2025-03-20

**Authors:** Robert Kwiatkowski, Anna M. Kłeczek, Jadwiga Gabor, Natalia Brzezińska, Andrzej S. Swinarew

**Affiliations:** 1 Radiotherapy Department, Katowice Oncological Center, 40-074 Katowice, Poland; 2 Faculty of Science and Technology, University of Silesia, 41-500 Chorzów, Poland; 3 Institute of Sport Science, The Jerzy Kukuczka Academy of Physical Education, 40-065 Katowice, Poland

**Keywords:** prostate cancer, radiotherapy, metastases, oligometastatic disease, multimodal treatment

## Abstract

**Simple Summary:**

Prostate cancer is a common disease in men. In some cases, it spreads to other parts of the body, making it more challenging to treat. Traditionally, treatment for metastatic prostate cancer has focused on easing symptoms rather than achieving remission. However, recent advances suggest that combining different treatments, including radiotherapy, may improve outcomes. This study explores a treatment plan that includes hormone therapy, chemotherapy, and targeted radiotherapy to both the primary tumor and metastatic sites. The results showed that this approach led to complete remission, with no signs of active cancer remaining. These findings suggest that radiotherapy should be considered part of a comprehensive treatment strategy for metastatic prostate cancer.

**Abstract:**

**Background/Objectives**: Prostate cancer is one of the most prevalent cancers among men, with a significant proportion progressing to metastatic disease. Traditional treatments for metastatic prostate cancer have primarily been palliative, focusing on symptom relief. However, recent advances in radiotherapy have shown promise in improving outcomes for these patients. **Methods**: This study presents a modern treatment plan for extensive metastatic prostate cancer. Pre-treatment imaging revealed extensive lymph node metastases and high metabolic activity in the prostate. The treatment regimen included bicalutamide, androgen deprivation therapy with leuprorelin, and six cycles of docetaxel chemotherapy, followed by a targeted radiotherapy regimen aimed at both the primary tumor and metastatic lymph nodes. **Results**: Following the comprehensive radiotherapy regimen, the patient’s PSA level dropped below the edge of detection, indicating complete biochemical remission. Follow-up imaging and clinical assessments confirmed the absence of active metastatic sites. **Conclusions**: The findings support the integration of radiotherapy into comprehensive treatment plans for metastatic prostate cancer, demonstrating that radiotherapy can achieve complete remission even in patients with extensive metastatic disease. This suggests a need for re-evaluating traditional approaches and exploring more personalized, multimodal treatment strategies. Enhanced imaging techniques, such as PET/PSMA scans, play a crucial role in accurately targeting metastatic sites, enabling more effective and individualized treatment.

## 1. Introduction

Prostate cancer is one of the most prevalent cancers among men globally, accounting for a significant proportion of cancer diagnoses and deaths. According to the World Health Organization (WHO), prostate cancer is the second most common cancer in men, with approximately 1.4 million new cases diagnosed worldwide in 2022 [1] and more than 1.5 million cases predicted in 2025 [2]. In the United States alone, the American Cancer Society estimates that about 313,780 new cases of prostate cancer will be diagnosed in 2025, and around 35,770 men will die from the disease [3]. These data highlight the critical need for effective treatments, particularly for advanced stages of the disease, where the prognosis is typically poorer.

Historically, the treatment of metastatic prostate cancer, wherein the cancer has advanced beyond the prostate gland to various parts of the body, has been predominantly palliative, with little influence in terms of survival. This approach aims to relieve symptoms and improve quality of life rather than cure the disease. Standard treatments for metastatic prostate cancer have included androgen deprivation therapy (ADT), chemotherapy, and palliative radiotherapy in the case of symptomatic disease. While these treatments can be effective in managing symptoms and slowing disease progression, they often do not offer long-term control or potential for cure.

For patients with localized prostate cancer, the treatment strategy is more aggressive, often involving surgical resection (prostatectomy) or radiotherapy as an alternative treatment and in cases with enhanced risk of recurrences.

Modern diagnostic modalities such as multiparametrical MR and PET/PSMA have demonstrated better accuracy in detecting both local and metastatic types of cancer. The advent of modern radiotherapy techniques and the better visualization of targets allow for the precise delivery of beams to very small lesions while sparing surrounding structures. This has significantly enhanced the efficacy of these treatments, providing better tumor control with fewer side effects. However, the application of radiotherapy in metastatic prostate cancer has traditionally been limited due to concerns about its effectiveness and potential adverse effects [4].

Simultaneously, there has been significant development in systemic treatments for locally advanced and diffused prostate cancer. Available options include inhibitors of androgen receptors, chemotherapy, and immunotherapy, which can be utilized in various lines of treatment [5].

Moreover, the role of the tumor microenvironment and inflammatory mediators in prostate cancer progression and treatment resistance has gained increasing attention. Cytokines, a diverse group of signaling molecules, play an important role in modulating the immune response and tumor behavior. In prostate cancer, pro-inflammatory cytokines such as interleukin-6 (IL-6), interleukin-8 (IL-8), or transforming growth factor-beta (TGF-β) have been implicated in disease progression and resistance to therapy [6]. IL-6, in particular, is known to activate the STAT3 signaling pathway, promoting tumor growth, survival, and resistance to apoptosis. Similarly, IL-8 has been associated with increased angiogenesis and metastatic potential in prostate cancer cells. Beyond their role in tumor progression, cytokines contribute to radio-resistance by modulating DNA damage repair mechanisms, promoting survival signaling pathways, and influencing the inflammatory response post radiotherapy. IL-1, for example, has been identified as a key factor in enhancing the resistance of cancer cells to ionizing radiation, potentially reducing the efficacy of radiotherapy [7]. Given the significant impact of cytokines on both prostate cancer progression and therapeutic response, targeting these pathways has emerged as a potential way to enhance radiotherapy outcomes. The modulation of cytokine activity, whether through direct inhibition or combination approaches, could improve radiosensitivity and provide better disease control for patients with advanced prostate cancer.

Advancements in radiotherapy, along with a better understanding of prostate cancer biology, have led to significant changes in the treatment of metastatic disease. The concept of oligometastatic disease, where cancer has spread to a limited number of sites (typically defined as 1–5 metastases), has emerged as a distinct clinical entity [8]. In these cases, targeted radiotherapy to metastatic sites, known as metastasis-directed radiotherapy (MDRT), can offer significant benefits, including prolonged survival time and improved quality of life.

Several key clinical trials have investigated the role of radiotherapy in both oligometastatic and polymetastatic prostate cancer. The HORRAD trial [9,10,11] explored the addition of local radiotherapy to ADT in patients with metastatic prostate cancer, revealing potential survival benefits in patients with a lower metastatic burden. The STAMPEDE trial [11,12,13,14], a large, multi-arm study, demonstrated that radiotherapy to the primary tumor site improves overall survival in men with newly diagnosed metastatic prostate cancer and a low metastatic burden. Similarly, the ORIOLE [15,16], STOMP [17,18], and SABR-COMET [19,20,21] trials have provided evidence supporting the use of stereotactic ablative body radiotherapy (SABR) for oligometastatic disease, showing high local control rates and minimal toxicity. The ARTO trial [22] investigated the combination of stereotactic body radiation therapy (SBRT) with abiraterone acetate in oligometastatic castrate-resistant prostate cancer, demonstrating significant benefits with improved biochemical response and progression-free survival.

Furthermore, the ongoing PEARLS trial [23], a phase II/III multicenter randomized controlled trial, aims to evaluate the safety and potential benefits of moderately fractionated extended field intensity-modulated radiotherapy (IMRT) in node-positive prostate cancer, potentially advancing treatment options for this patient population.

The findings suggest that modern radiotherapy techniques can be integrated into comprehensive treatment plans for metastatic prostate cancer, potentially transforming outcomes for patients with advanced disease. The advancements in radiotherapy for prostate cancer treatment underscore the importance of re-evaluating traditional approaches to metastatic disease. While the benefits of radiotherapy in oligometastatic prostate cancer are well documented, research also indicates that patients with a higher number of metastatic sites—beyond the typical oligometastatic threshold of five—can still derive significant benefits from radiotherapy [24]. This paper presents an example of one such case treated at the hospital in Katowice, demonstrating the potential of radiotherapy to improve outcomes even in patients with more extensive metastatic disease.

## 2. Materials and Methods

**Figure 1:**
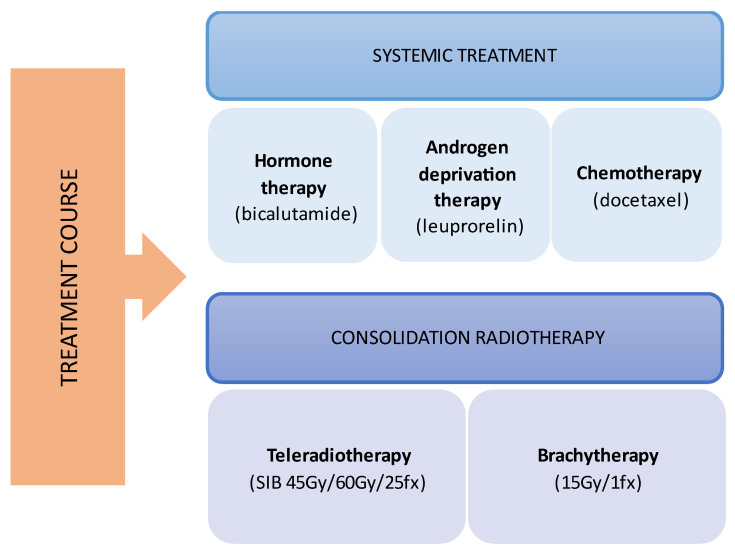
Multimodal treatment course.

A 57-year-old male patient with metastatic prostate cancer, initially diagnosed with a highly elevated PSA level, presented for treatment (Figure 1) at the hospital in Katowice. Upon diagnosis, the patient underwent a comprehensive medical evaluation, including a prostate biopsy, which led to the initiation of bicalutamide therapy. Further imaging studies, including a CT scan of the abdomen and pelvis and bone scintigraphy, were performed to assess the extent of the disease.

To better understand the distribution and activity of the cancer, a PET/PSMA scan was conducted. This imaging modality provided detailed information on the metastatic spread and the primary tumor’s metabolic activity. Following these diagnostic procedures, the patient was treated with androgen deprivation therapy using leuprorelin.

Given the extensive spread of the disease to lymph nodes, the patient was also administered six cycles of docetaxel chemotherapy. Post-chemotherapy imaging was conducted to evaluate the response to treatment and guide further therapeutic decisions.

Despite systemic therapy, the patient exhibited signs of minimal residual disease, and his PSA levels remained elevated, indicating persistent disease activity. Considering the comprehensive diagnostic findings and the patient’s treatment history, a decision was made to proceed with radiotherapy as a consolidation therapy.

**Figure 2:**
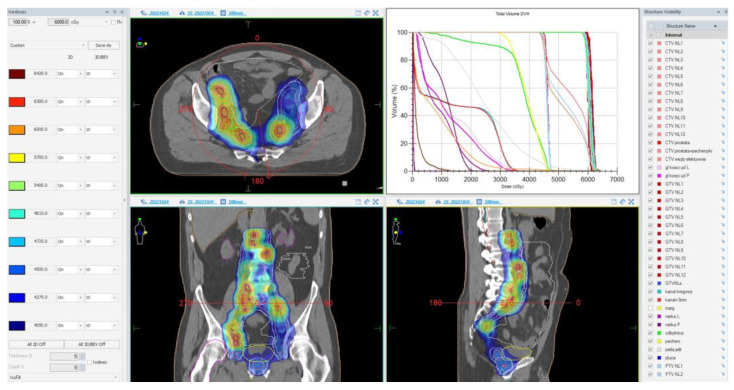
Radiotherapy treatment planning—SIB 45 Gy/60 Gy/25 fx. The treatment plan is visualized across axial, coronal, and sagittal planes, with a corresponding dose–volume histogram illustrating the spatial distribution of radiation doses and treatment field coverage. The color spectrum represents different dose levels, with red indicating the highest dose areas and blue indicating the lowest dose areas. The plan was designed to maximize tumor coverage while sparing healthy tissue.

**Figure 3:**
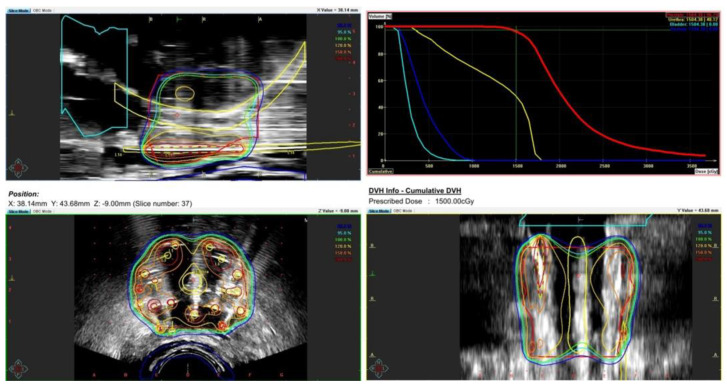
Radiotherapy treatment planning—boost BRT 15 Gy/1 fx. The treatment plan outlines the prostate-focused brachytherapy boost aiming for optimized tumor control while minimizing toxicity. The accompanying dose–volume histogram illustrates the dose distribution within the prostate, urethra, bladder, and rectum.

During the planning phase of the radiotherapy treatment course, a PET/CT scan was performed to delineate the metastatic sites accurately and guide radiotherapy planning. The radiotherapy regimen included teleradiotherapy targeting the prostate and affected lymph nodes with radical intent, utilizing a simultaneous integrated boost (SIB) approach with 45 Gy/60 Gy delivered in 25 fractions (Figure 2). Additionally, a high-dose–rate (HDR) brachytherapy boost of 15 Gy was administered in a single fraction to the prostate to achieve maximum local control (Figure 3).

Throughout the treatment process, the patient’s clinical status and response to therapy were closely monitored, with regular follow-up assessments to evaluate the effectiveness of the interventions and manage any treatment-related adverse events.

## 3. Results

**Figure 4:**
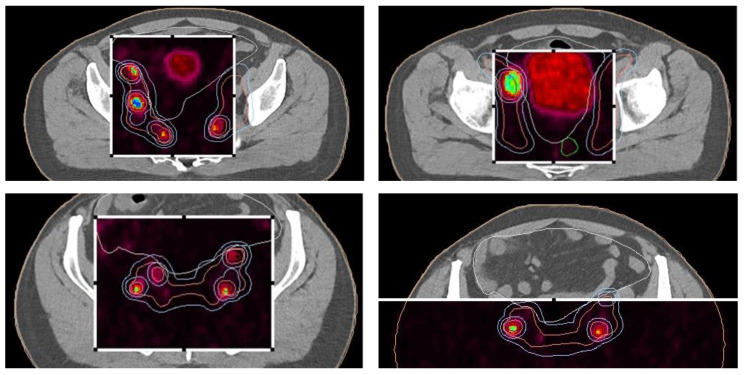
PET/PSMA scan of the patient showing metastatic sites in the prostate and lymph nodes. The scan highlights the primary tumor site in the prostate and identifies metastatic lymph nodes, illustrating the spread of the disease.

The patient’s initial PSA level was 280 ng/mL at diagnosis, and a prostate biopsy confirmed adenocarcinoma with a Gleason score of 7 (4 + 3), WHO Grade group 3. CT imaging of the abdomen and pelvis revealed extensive metastasis to the pelvic lymph nodes, including the obturator, external iliac, and common iliac nodes, as well as involvement in the para-aortic space. A bone scintigraphy showed no signs of bone metastasis. Subsequent PET/PSMA imaging (Figure 4) identified pathological tracer uptake in the majority of the prostate tissue bilaterally, with an SUV max of 19.2 and a visual score of 3, E-PSMA 5. Numerous pelvic lymph nodes, including the external, internal, and common iliac nodes bilaterally, showed an SUV max of 36.2 and a short-axis diameter up to 22 mm, visual score 3, E-PSMA 5. Additionally, retroperitoneal lymph nodes on both sides showed SUV max values up to 32 and short-axis diameters up to 9 mm, visual score 3, E-PSMA 5. The distribution of the radiotracer was otherwise physiological.

Based on these findings, the patient was classified as having stage cT2N1M1a prostate cancer with metabolically active disease in the prostate and extensive lymph node involvement. After receiving six cycles of docetaxel chemotherapy, his PSA level decreased from 118 ng/mL to 6.81 ng/mL. However, subsequent CT imaging of the abdomen and pelvis continued to show extensive lymph node metastases.

**Figure 5:**
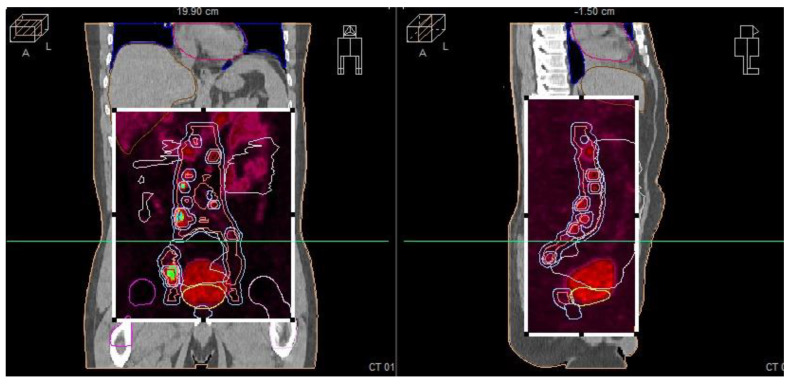
Radiotherapy treatment planning—PET-CT—showing metastatic sites in the prostate and lymph nodes.

Pre-radiotherapy PET-CT imaging (Figure 5) showed extensive lymph node metastases and high metabolic activity in the prostate. Following radiotherapy, the patient’s PSA level dropped below the edge of detection, indicating complete biochemical remission. This significant response was confirmed through follow-up imaging and clinical assessments.

## 4. Discussion

The presented case highlights the potential for radiotherapy to induce significant clinical responses even in patients with extensive metastatic prostate cancer. The initial diagnosis revealed a high PSA level and a Gleason score of 7 (4 + 3), indicating a high-grade tumor with a substantial metastatic burden. The comprehensive diagnostic workup, including CT, bone scintigraphy, and PET/PSMA imaging, confirmed widespread lymph node involvement without bone metastases. This extensive lymph node involvement typically indicates a poorer prognosis, necessitating aggressive treatment.

Despite initial systemic therapy with bicalutamide and subsequent androgen deprivation therapy using leuprorelin, the patient’s disease exhibited minimal residual activity, as evidenced by persistently elevated PSA levels. The addition of docetaxel chemotherapy reduced the PSA level but did not eliminate lymph node metastases. This partial response underscored the need for a multimodal approach to achieve better disease control.

The decision to proceed with MDRT as a consolidation therapy was based on the persistence of metabolically active disease post chemotherapy. The radiotherapy regimen included both external-beam radiotherapy (EBRT) and HDR brachytherapy, targeting the primary tumor and metastatic lymph nodes with precise dosages aimed at maximizing local control. This comprehensive approach is supported by emerging evidence from clinical trials, which suggests that targeted radiotherapy can improve outcomes even in patients with a significant metastatic burden.

The post-radiotherapy outcomes in this case were notable, with the patient’s PSA level dropping below the detection threshold, indicating complete biochemical remission. This result is significant, demonstrating that aggressive radiotherapy can achieve complete remission in metastatic prostate cancer, even when the metastatic burden exceeds the oligometastatic threshold of five lesions. This finding aligns with the evolving understanding that metastatic prostate cancer is not a homogenous entity and that a subset of patients with extensive metastases may still benefit from localized treatments. However, it is crucial to acknowledge that this study is based on a single patient, and extrapolating these findings to a broader population remains uncertain. While this case illustrates the potential role of metastasis-directed radiotherapy beyond the conventional oligometastatic threshold, its applicability to other patients requires further validation.

Importantly, this case illustrates that the decision for or against metastasis-directed radiotherapy should not be based solely on a fixed number of metastases but rather on the ability to identify and effectively target all metastatic sites. In this case, PET/PSMA imaging identified numerous metastatic lymph nodes, including the external, internal, and common iliac nodes bilaterally with a short-axis diameter of up to 22 mm and SUV max values reaching 36.2, as well as retroperitoneal lymph nodes. Despite the high number of metastatic sites, the treatment was successful, resulting in significant clinical benefits. These findings challenge the conventional boundary of a maximum of five metastases for oligometastatic disease and suggest that a more individualized approach can yield significant clinical benefits.

The potential of stereotactic body radiotherapy to precisely target small fields of metastatic disease offers an additional therapeutic advantage. SBRT, with its high precision and ability to deliver high doses of radiation to small, well-defined areas, can act as an immunomodulator, stimulating the immune system and potentially enhancing the effectiveness of other treatments such as immunotherapy.

However, the risks and benefits of MDRT must be carefully balanced in all metastatic patients. Normal tissue toxicity is dependent on the anatomic location of the disease receiving therapy, and the potential for side effects must be weighed against the expected benefits. Factors such as quality of life, treatment-free interval, and progression-free survival (often used to evaluate the effectiveness of oncological treatments) should be considered when determining the appropriateness of MDRT for individual patients.

Beyond treatment-related toxicities, biological factors such as IL-1 could also play a role in determining radiotherapy outcomes, with evidence linking IL-1 to radio-resistance in cancer therapies [7]. IL-1 can promote tumor progression, enhance DNA damage repair mechanisms in cancer cells, and contribute to an immunosuppressive tumor microenvironment. These factors collectively reduce the effectiveness of radiotherapy by allowing cancer cells to evade radiation-induced apoptosis. Furthermore, IL-1 has been implicated in the development of cardiovascular complications, which are a significant concern in patients receiving radiotherapy, particularly those with pre-existing risk factors. Targeting IL-1 with blocking agents has emerged as a promising strategy to enhance the anticancer efficacy of radiotherapy while minimizing adverse cardiovascular effects. IL-1 inhibitors, such as canakinumab, can mitigate the inflammatory responses induced by radiation, thereby reducing radiation-induced toxicity and improving tumor control. Integrating IL-1 blockade into treatment regimens for metastatic prostate cancer may offer a dual benefit—enhancing the radiosensitivity of tumors and reducing the long-term cardiovascular risks associated with radiotherapy.

In this context, the importance of a multidirectional approach becomes evident. The current standard in treating prostate cancer involves the intensification of systemic treatment, integrating radiation therapy as a crucial component of a multidisciplinary treatment strategy. Modalities such as hormone therapy, androgen deprivation therapy, chemotherapy, immunotherapy, and radiotherapy are often combined to exploit their synergistic effects, providing more effective results than any single treatment, especially for disseminated tumors. In the presented case, the patient initially received hormone therapy, followed by chemotherapy due to the advanced stage of the disease, and, finally, radiation therapy. This sequential approach led to a significant reduction in PSA levels, ultimately resulting in complete biochemical remission and no detectable active metastatic sites. Each stage of treatment contributed to the overall therapeutic effect, emphasizing the value of multimodal therapy.

## 5. Conclusions

The presented medical case illustrates the potential for radiotherapy to achieve significant clinical benefits in patients with extensive metastatic prostate cancer. The patient’s response to a comprehensive radiotherapy regimen, despite a high initial metastatic burden, underscores the importance of considering radiotherapy as a viable treatment option in the advanced stages of the disease. The complete biochemical remission achieved post radiotherapy highlights the role of radiotherapy in achieving disease control and potentially improving survival outcomes.

These findings contribute to the growing body of evidence supporting the use of radiotherapy in metastatic prostate cancer and suggest that even patients with a higher number of metastatic sites can benefit from targeted radiotherapy. The traditional threshold for defining oligometastatic disease is based on the premise that effective treatment of all metastatic sites can improve outcomes. However, this case suggests that the benefits of metastasis-directed radiotherapy may extend beyond this conventional boundary, reinforcing the need for a more individualized treatment approach.

This case demonstrates that, when all metastatic sites can be identified and treated, patients with more than five metastatic sites may still derive significant benefits from metastasis-directed radiotherapy. Advancements in imaging techniques, including CT, MRI, and PET/PSMA scans, have significantly improved the accuracy of detecting and visualizing metastatic sites, allowing for the precise targeting of radiotherapy and ensuring more effective and individualized therapeutic strategies.

The use of stereotactic body radiotherapy for targeting small fields of metastatic disease offers high precision and the potential to act as an immunomodulator, stimulating the immune system and enhancing the efficacy of systemic treatments such as immunotherapy. Additionally, emerging evidence suggests that cytokines could play a crucial role in modulating the response to radiation treatment and may help mitigate long-term cardiovascular risks associated with systemic inflammation in patients with cancer.

However, the potential benefits of radiotherapy must be balanced against the risks of treatment-related adverse events and their impact on the patient’s quality of life. Factors such as the location of the disease and its effects on normal tissues highlight the need for precise targeting and personalized treatment planning, essential for optimizing outcomes.

Integrating hormone therapy, chemotherapy, and radiotherapy leverages the cumulative benefits of a multidirectional treatment approach. This comprehensive strategy not only targets cancer more effectively but can also significantly prolong the time to disease progression, thereby improving long-term survival outcomes for patients with advanced metastatic prostate cancer.

Future research should continue to explore the optimal integration of radiotherapy with systemic therapies and investigate the potential benefits in larger, more diverse patient populations. This case underscores the need for personalized treatment approaches, taking into account the individual patient’s disease characteristics and response to prior therapies, to maximize the therapeutic benefits of radiotherapy in metastatic prostate cancer.

## Data Availability

Research data are stored in an institutional repository and will be shared upon request to the corresponding author.
